# Structural Characterization and Immunomodulatory Activity of a Polysaccharide From *Ipomoea batatas* L. Vine: Activation of Macrophage

**DOI:** 10.1002/fsn3.72530

**Published:** 2026-11-03

**Authors:** Yuqi Shi, Bo Si, Yu Shi, Wenxin Geng, Dunchi Xiao, Zhixiang Yuan, Liwen Li

**Affiliations:** ^1^ Changzhou Institute of Materia Medica co., Ltd. Changzhou China; ^2^ Key Laboratory of Integrated Management of Crop Diseases and Pests (Nanjing Agricultural University) Ministry of Education Nanjing China; ^3^ School of Materials Science and Engineering Changzhou University Changzhou China; ^4^ Key Laboratory of Resource Biology and Biotechnology in Western China Northwest University, Ministry of Education Xi'an China; ^5^ College of Life Sciences Northwest University Xi'an China

**Keywords:** macrophage, polarization, polysaccharide, structural characterization, sweet potato

## Abstract

Sweet potato vines, which have long been consumed in East Asia, are often underutilized despite being a nutritious functional food. In this study, a pectin named IBPS‐3A was isolated and purified from sweet potato vines. Comprehensive structural characterization using FT‐IR, monosaccharide composition analysis, methylation analysis, and 1D/2D NMR spectroscopy (COSY, HSQC, TOCSY, HMBC, and NOESY) identified IBPS‐3A as an acidic heteropolysaccharide with a molecular weight of approximately 56 kDa, primarily composed of a backbone containing →4)‐α‐D‐Gal*p*A‐(1 → and →2,4)‐α‐L‐Rha*p*‐(1 → linkages with arabinogalactan side chains. Further investigation revealed that IBPS‐3A dose‐dependently induced M1 polarization in RAW264.7 macrophages, stimulating the secretion of TNF‐α, IL‐6, and NO and subsequently inhibiting co‐cultured B16 melanoma cell viability. Mechanistically, Toll‐like receptor (TLR) 4 and TLR2 represent potential receptors contributing to the immunostimulatory effects of IBPS‐3A leading to subsequent activation of the NF‐κB and MAPK (p38) signaling cascades. Collectively, these findings indicate that IBPS‐3A is a bioactive polysaccharide from sweet potato vines and highlight its potential for immunomodulatory applications.

## Graphical abstract

IBPS‐3A, an acidic heteropolysaccharide purified from sweet potato vines, was structurally characterized to possess a backbone consisting of →4)‐α‐D‐Gal*p*A‐(1→ and →2,4)‐α‐L‐Rha*p*‐(1→ linkages with arabinogalactan side chains. Further investigation revealed that IBPS‐3A induced M1 polarization in RAW264.7 macrophages, stimulating the secretion of TNF‐α, IL‐6, and NO and subsequently inhibiting the viability of co‐cultured B16 melanoma cells, rather than acting directly on B16 cells. Mechanistically, Toll‐like receptor (TLR) 4 and TLR2 represent potential receptors contributing to the immunostimulatory effects of IBPS‐3A. This work demonstrates that IBPS‐3A is a bioactive polysaccharide from sweet potato vines and highlights its potential for immunomodulatory applications.

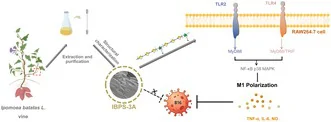

## 1 Introduction

Sweet potato ( *Ipomoea batatas* L.) is a high‐yield, adaptable food crop of the Convolvulaceae family. Originating in South America, it is now widely grown in tropical, subtropical, and temperate regions (Yang et al. 2018; Cai et al. 2022). Sweet potato is rich in carbohydrates, protein, folate, vitamins, and flavonoids, which may contribute to enhanced immune function and disease prevention (Alam 2021; Laveriano‐Santos et al. 2022). Its stems and leaves are similarly nutrient‐dense and contain various bioactive compounds, garnering increasing attention (Yang et al. 2019; Zhang et al. 2025). Sweet potato vines have a long history of being consumed in East Asia and, according to traditional Chinese medicine theory, are believed to have pharmacological effects such as dispelling wind and unblocking collaterals, promoting blood circulation and dispersing stasis, as well as reducing swelling and alleviating pain. With ongoing research, small‐molecules compounds such as flavonoids and vitamins have been isolated from sweet potato vines, and their immunomodulatory and antioxidant functions have been confirmed (Adu Donyina et al. 2025). However, studies on the active components of sweet potato vines remain far less extensive than those on sweet potato roots, particularly with respect to structurally complex biomacromolecules such as pectic polysaccharides.

Pectic polysaccharides are a class of acidic heteropolysaccharides that are widely distributed within plant primary plant cell walls and middle lamellae (Lara‐Espinoza et al. 2018). As the major active components of plants, they have significant applications in health, pharmaceutical, and food sectors (Rosales and Fabi 2023; Kalita et al. 2025). The structural composition of pectin differs depending on the plant source, growth environment, and extraction approach. The structure of pectin primarily includes four basic domains, namely homogalacturonan (HG), rhamnogalacturonan‐I (RG‐I), rhamnogalacturonan‐II (RG‐II), and xylogalacturonan (XGA), with XGA reported in specific plants (Bai and Gilbert 2022; Sanhueza et al. 2024). In addition, there are a few specialized structural domains, such as apiogalacturonan (AGA) (Wu et al. 2024). With deepening understanding of the pectin structure, its biological activities have attracted increasing attention. Pectic polysaccharides display a broad spectrum of pharmacological effects, including antioxidant, hypoglycemic, immunomodulatory, antitumor, anti‐inflammatory, and antiviral properties (Xiang et al. 2024). For instance, Vogt et al. (2016) found that lemon pectin can exert immunomodulatory and epithelial protective effects by stimulating Toll‐like receptors (TLRs) to activate the nuclear factor kappa B (NF‐κB) and AP‐1 signaling axes. Research by Zou et al. (2014) revealed that the RG‐I‐type pectin from *Codonopsis pilosula* activates the complement system via its arabinogalactan side chains and stimulates B and T cell activation, thereby enhancing the release of anti‐inflammatory mediators. Zhang et al. (2012) confirmed that the ginseng pectin WGPA‐2‐RG enhances lymphocyte proliferation and macrophage phagocytic function, demonstrating clear immunomodulatory activity. These findings indicate that the fine structural features of pectin are closely associated with its immunomodulatory activity, highlighting the importance of elucidating the structure–activity relationship and the underlying mechanisms of pectin polysaccharides. However, the structural characterization of pectic polysaccharides derived from sweet potato vines has not yet been stematically performed, and the structure–activity relationship between their structural features and immunomodulatory activities remains unknown.

Immunotherapy has recently emerged as a promising avenue for cancer treatment. Macrophage‐targeted therapeutic strategies primarily focus on inhibiting the pro‐tumor characteristics of M2‐like macrophages, promoting M1‐like macrophage polarization, and reprogramming macrophages from an M2‐like toward an M1‐like phenotype (Strizova et al. 2023). Certain chemotherapeutic agents, such as doxorubicin, can also enhance tumor immunogenicity while inducing M1 polarization in macrophages (Guo et al. 2013). Importantly, several pectic polysaccharides have been reported to promote M1 polarization. For example, the pectic polysaccharide PEP‐1 from *Phyllanthus emblica* promotes the M2‐to‐M1 phenotypic conversion of macrophages, and conditioned medium from PEP‐1‐treated M2 macrophages induces apoptosis in Hepa1‐6 cells (Zong et al. 2025). Similarly, GJP70‐0.2 from gardenia fruits enhances the proliferation and phagocytic activity of RAW264.7 macrophages and promotes M1 polarization through the NF‐κB and JAK/STAT signaling pathways (Guo et al. 2025). However, it remains unclear whether pectic polysaccharides from sweet potato vines exert immunomodulatory effects through macrophage polarization.

In this study, a polysaccharide IBPS‐3A was obtained and purified from sweet potato vines, and its structural features were systematically characterized. The immunomodulatory effect of the polysaccharide, particularly its ability to induce M1 polarization in RAW264.7 macrophages, was investigated. Our results demonstrate that this pectic polysaccharide promotes macrophage M1 polarization primarily through TLR4 and TLR2 receptors, subsequently triggering downstream NF‐κB and mitogen‐activated protein kinase (MAPK) cascades. This study establishes a foundation for utilizing sweet potato vine polysaccharides as functional foods for immunomodulation.

## 2 Materials and Methods

### 2.1 Materials and Reagents

Sweet potato vines were provided by Nanjing Agricultural University. After removal of the leaves, they were dried at 60°C for later use. Cellulose DEAE‐52 resin was purchased from Beijing Solarbio Science & Technology Co. Ltd., Superdex 200 Prep Grade was obtained from GE Healthcare Life Sciences, and the antibodies used in the experiments were sourced from ABclonal.

### 2.2 Isolation and Purification

Dried sweet potato vines were ground into a powder. After mixing an appropriate amount of the powder with 20 volumes (w/v) of distilled water, the mixture was heated at 90°C for 3 h. Three portions of absolute ethanol were added to the supernatant, and the mixture was thoroughly mixed. The resulting solution was left at 4°C overnight to precipitate the polysaccharides (Yuan et al. 2020). The resulting pellet obtained after centrifugation was redissolved in distilled water and deproteinized using the Sevag method (Yuan et al. 2020). The residual Sevag reagent was removed by rotary evaporation and concentration (Li et al. 2024). Subsequently, three portions of absolute ethanol were mixed to precipitate the alcohol precipitation. After redissolution, the precipitate was lyophilized to obtain the crude polysaccharide, referred to as IBPS.

An appropriate amount of IBPS was solubilized in distilled water and purified using a DEAE‐52 anion exchange column. Elution was performed using NaCl solutions of varying concentrations and the total carbohydrate content in the collected fractions was measured using a phenol‐sulfuric acid assay (Dubois et al. 1956).

Subsequently, IBPS‐3 was solubilized in distilled water and fractionated using a Superdex G‐200 column. Elution was performed using distilled water at a constant flow rate. The eluent was collected, and the total carbohydrate content in each fraction was measured via the phenol‐sulfuric acid method, allowing the construction of an elution curve (Wei et al. 2021). The polysaccharide fraction corresponding to the peak in the profile was collected, concentrated, and processed for alcohol precipitation by adding absolute ethanol to reach a final concentration of 50%, followed by overnight incubation. The obtained precipitate was redissolved and lyophilized, and the purified fraction was designated as IBPS‐3A (Figure 1A).

**FIGURE 1:**
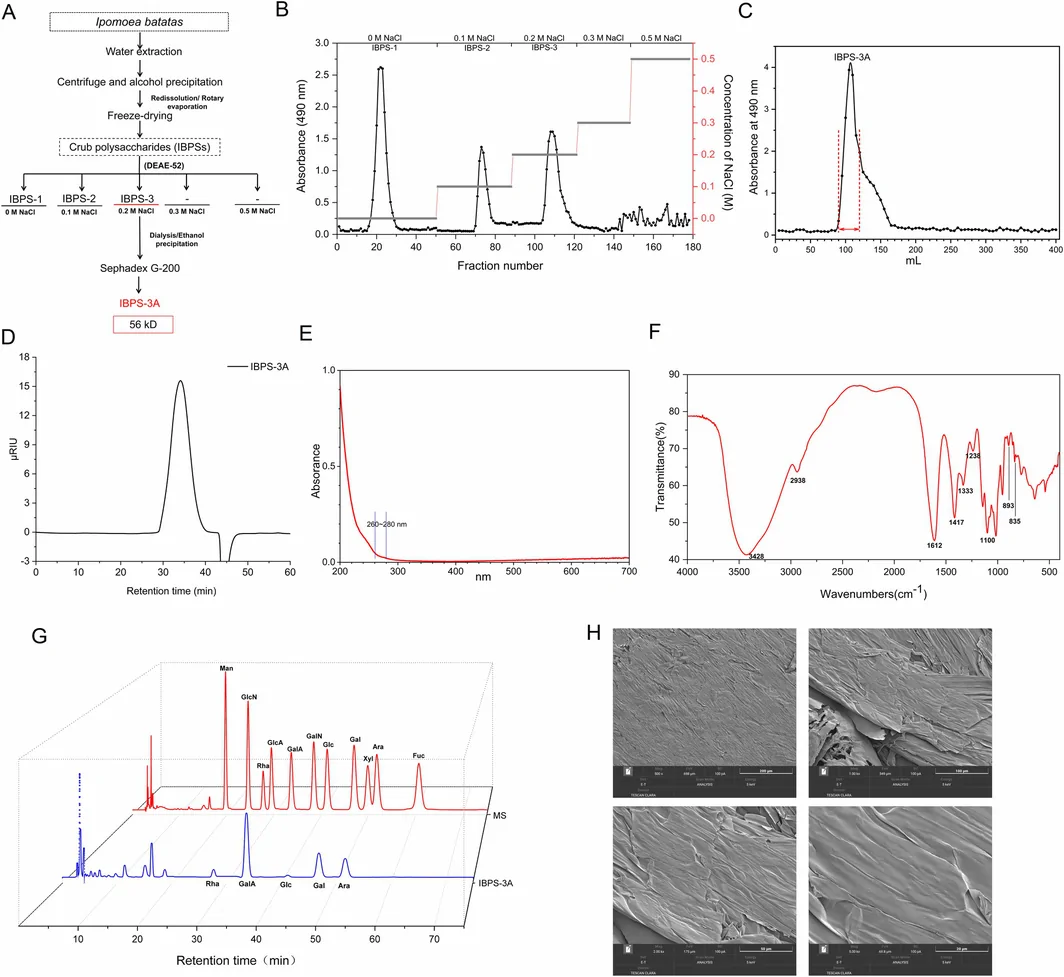
Isolation and purification of IBPS‐3A. (A) Experimental protocol of extraction and purification of the polysaccharide IBPS‐3A from *Ipomoea batatas* L. vine; (B) The elution curve of IBPS by DEAE‐52 cellulose; (C) The elution curve of IBPS‐3A by Superdex G‐200 chromatography; (D) The HPGPC chromatogram of IBPS‐3A; (E) The UV–Vis spectrum of purified IBPS‐3A; (F) The Fourier‐transform infrared (FT‐IR) spectrum of IBPS‐3A; (G) Monosaccharide composition analysis of IBPS‐3A; (H) Scanning electron microscopy (SEM) of IBPS‐3A.

### 2.3 Analysis of Chemical and Monosaccharide Composition

The total carbohydrate content of IBPS‐3A was measured using the phenol‐sulfuric acid method, whereas the protein content was measured using the Coomassie Brilliant Blue method (Blakesley and Boezi 1977). A spectrophotometer was used to scan IBPS‐3A across 200–700 nm wavelengths to assess the presence of proteins or nucleic acids. The level of endotoxin in IBPS‐3A was measured by a LAL assay using an endotoxin assay kit (Zhanjiang A&C Biological Ltd., China) according to the manufacturer's instructions. The molecular weight (Mw) and distribution of IBPS‐3A were analyzed using high‐performance gel permeation chromatography (HPGPC) with a series connection of Ohpak SB‐804 HQ and Ohpak SB‐806 M HQ columns. IBPS‐3A was dissolved in 0.05 mol/L NaCl and analyzed at 0.5 mL/min with a 30 μL injection volume. A refractive index detector (RID) was used. Calibration of the system was performed using Pullulan standards of defined molecular weights.

To determine the monosaccharide composition of IBPS‐3A, high‐performance liquid chromatography (HPLC) was employed. The hydrolysis of a precisely weighed sample of IBPS‐3A was performed using 2 mol/L trifluoroacetic acid (TFA) at 110°C for 4 h (Liu et al. 2019). The hydrolysate was then dried under a nitrogen stream. After dissolution in methanol, the residue was re‐dried under nitrogen; this process was repeated two to three times to thoroughly eliminate the residual TFA. The resulting residue was dissolved in distilled water, filtered, and subjected to HPLC analysis (Imran et al. 2025).

Chromatographic separation was carried out using an Agilent ZORBAX Eclipse XDB‐C18 column (4.6 × 250 mm, 5 μm) maintained at 30°C. The isocratic elution employed a solvent system consisting of acetonitrile and phosphate buffer in a 17:83 volume ratio. The phosphate buffer contained 12 g/L potassium dihydrogen phosphate, adjusted to pH 6.8 using 2 mol/L NaOH. The flow rate was set at 0.8 mL/min, and the absorbance was measured at 250 nm. The injection volume was 10 μL.

### 2.4 Fourier Transform Infrared Spectroscopy and Scanning Electron Microscopy Analysis

A mixture of approximately 2 mg IBPS‐3A and 200 mg KBr was prepared and ground thoroughly in a mortar to form a homogeneous sample. The sample was then analyzed via a Fourier‐transform infrared (FT‐IR) spectrometer, with scanning performed across 4000–400 cm^−1^ wavenumbers (Xiong et al. 2021).

For scanning electron microscopy (SEM), IBPS‐3A was sputter‐coated with gold and examined under a Nova NanoSEM450 microscope. The imaging was performed at an accelerating voltage of 10 kV (Correia et al. 2020).

### 2.5 Methylation Analysis

IBPS‐3A is an acidic polysaccharide, which requires reduction of its uronic acids prior to methylation analysis. In short, 5 mg of IBPS‐3A was solubilized in distilled water, followed by adding 200 μL of 2 mol/L MES solution along with 500 μL of 200 mg/mL carbodiimide. The mixture was then incubated under ambient conditions for 2 h. Subsequently, 1 mL of 2 mol/L imidazole and 1 mL of 70 mg/mL NaBD_4_ were added, and the mixture was allowed to proceed under ambient conditions for an additional 3 h. The reaction was terminated by adding glacial acetic acid. The resulting sample was dialyzed against distilled water for 48 h and lyophilized to obtain the uronic acid‐reduced product (Xu et al. 2025).

Methylation was performed as described by Yuan et al. (2024). The methylated derivatives were analyzed using an Agilent 7890A‐5977B gas chromatography–mass spectrometry system (Agilent Technologies Inc., CA, USA). Separation was achieved on an HP‐5MS capillary column (30 m × 0.25 mm × 0.25 μm, Agilent J&W Scientific, Folsom, CA, USA). Mass spectrometry was performed using an EI ion source at 230°C. Data were recorded in SCAN mode across 50–600 m/z, controlled by the MassHunter workstation software (Huang et al. 2022).

### 2.6 Nuclear Magnetic Resonance (NMR) Analysis

The deuterated IBPS‐3A sample was dissolved in 0.5 mL of D_2_O and analyzed using an Agilent 800 MHz DD2 NMR spectrometer. One‐dimensional NMR spectra, including ^1^H‐NMR, ^13^C‐NMR, and DEPT‐135, were acquired. Furthermore, two‐dimensional NMR experiments, namely HSQC, COSY, TOCSY, HMBC, and NOESY, were performed. Data processing and analysis were conducted using the MestRe Nova software (version 14.2.3, Spain) (Yuan et al. 2024).

### 2.7 RAW264.7 Cell Polarization Assay

#### 2.7.1 RAW264.7 Cell Viability Determination

The macrophage cell line RAW 264.7 and mouse melanoma cell line B16 were obtained from the Chinese Academy of Sciences Cell Bank (Shanghai, China). RAW 264.7 and B16 cells were cultured in DMEM (high glucose) supplemented with 10% FBS (Gibco) and 1% penicillin (100 U/mL) and streptomycin (100 μg/mL) (Beyotime) in a humidified incubator at 37°C with 5% CO_2_.

RAW264.7 cells were loaded into 96‐well plates at 2 × 10^5^ cells/well and incubated under standard conditions. After 24 h, the culture medium was refreshed with medium supplemented with IBPS‐3A at 6.25–50 μg/mL, lipopolysaccharide (LPS) at 1 μg/mL, or blank medium (control group). Cells were further cultured for 24 h. Following this incubation, the supernatant was discarded, and each well received 100 μL of Cell Counting Kit‐8 (CCK‐8) solution. The plates were then kept at 37°C for 2 h, after which the absorbance was measured at 450 nm (Qi et al. 2025).

#### 2.7.2 Detection of Polarized Products in RAW264.7 Cells

RAW264.7 cells were loaded into 6‐well plates at 2 × 10^5^ cells/well, supplied with 2 mL of complete DMEM per well. After 24 h, the culture medium was refreshed with medium supplemented with IBPS‐3A at 6.25–50 μg/mL, LPS at 1 μg/mL, or blank medium (control group). The cells were then maintained for a further 24 h. Following incubation, the culture supernatants were collected by centrifugation (12,000 × g, 5 min) and subsequently used for the determination of tumor necrosis factor‐alpha (TNF‐α), interleukin‐6 (IL‐6), and nitric oxide (NO) levels.

The levels of TNF‐α and IL‐6 in the collected cell culture supernatants were quantified using the Beyotime Mouse TNF‐α ELISA Kit (PT512) and the Beyotime Mouse IL‐6 ELISA Kit (PI326), respectively. NO level in the supernatant was determined using the Beyotime Nitric Oxide Assay Kit (S0021S) (Yang et al. 2024).

The cells were rinsed three times with phosphate‐buffered saline (PBS) and subsequently lysed on ice for 30 min using RIPA lysis buffer supplemented with 1% PMSF, 1% protease inhibitor, and 1% phosphatase inhibitor, and vortexed every 5 min. After centrifugation, the supernatant was collected. Protein samples were loaded onto a 10% sodium dodecyl sulfate‐polyacrylamide gel electrophoresis (SDS‐PAGE) gel. After electrophoresis, the proteins were blotted onto a polyvinylidene difluoride (PVDF) membrane. After incubation with appropriate primary and secondary antibodies, protein bands were detected using an enhanced chemiluminescence (ECL) system, followed by quantification using ImageJ software (Su et al. 2020).

### 2.8 Conditioned Medium Cytotoxicity Assay on B16 Cells

RAW264.7 cells were loaded into 6‐well plates at 2 × 10^6^ cells/well. After 24 h of culture, the culture medium was refreshed with medium supplemented with IBPS‐3A at 6.25–50 μg/mL, LPS at 1 μg/mL, or blank medium (control group) devoid of both IBPS‐3A and LPS. The cells were maintained for a further 24 h. Subsequently, the culture media were harvested and centrifuged at 12,000 × g for 5 min. The supernatants collected from RAW264.7 cells treated with IBPS‐3A at 6.25–50 μg/mL were designated as conditioned media (CM).

B16 cells were loaded at 8 × 10^3^ cells/well in 96‐well plates containing 100 μL complete DMEM. After 24 h, the culture medium was replaced with 100 μL of treatment medium containing CM at 1.25%, 2.5%, 5%, 10%, 20%, 30%, 40%, or 50% (v/v); CM‐free complete DMEM was used as the control. These CM concentrations were selected based on preliminary experiments. After a further 24‐h incubation, supernatants were discarded. Subsequently, 100 μL of CCK‐8 reagent, prepared in complete DMEM, was introduced into each well. Following 2 h of incubation at 37°C, the absorbance was measured at 450 nm.

### 2.9 Co‐Culture System Experiment

A co‐culture experiment using RAW264.7 and B16 cells was conducted in a 24‐well plate employing Transwell inserts (8 μm pore size). RAW264.7 cells were loaded into the lower chamber of the 24‐well plate at densities of 2.5–8 × 10^5^ cells per well. B16 cells (0.5–1.5 × 10^5^ cells/well) were seeded into the upper Transwell chamber. The co‐culture system was then maintained for 24 h in complete medium supplemented with IBPS‐3A at 6.25–50 μg/mL, LPS at 1 μg/mL, or blank medium (control group) devoid of both IBPS‐3A and LPS. After incubation, supernatants were discarded. Subsequently, 100 μL of CCK‐8 solution, prepared in complete medium, was introduced into each well. After 2 h of incubation at 37°C, the absorbance of each well was determined at 450 nm.

### 2.10 TLR2 and TLR4 Inhibitor Assay

To investigate the involvement of TLR2 and TLR4 in IBPS‐3A‐induced macrophage activation, the TLR2 inhibitor C29 (MedChemExpress, Cat. No. HY‐100461) and the TLR4 inhibitor TAK‐242 (MedChemExpress, Cat. No. HY‐11109) were used. C29 and TAK‐242 were respectively dissolved in dimethyl sulfoxide (DMSO) to prepare 1 mM stock solutions for subsequent use.

RAW264.7 macrophages were seeded into 6‐well plates at 2 × 10^6^ cells/well and cultured for 24 h. The cells were then treated with C29 (50 μM), TAK‐242 (10 μM), or both for 2 h. After inhibitor pretreatment, the medium was removed, and the cells were treated with IBPS‐3A at 50 μg/mL for 24 h. Untreated cells served as the negative control, and cells treated with LPS (1 μg/mL) for 24 h served as the positive control. After treatment, the culture supernatants were collected to measure TNF‐α, IL‐6, and NO levels.

### 2.11 Western Blot Analysis

Cells were washed three times with PBS and lysed at 4°C for 30 min in RIPA lysis buffer supplemented with 1% PMSF, 1% protease inhibitor, and 1% phosphatase inhibitor, with vortexing every 5 min. The lysates were centrifuged at 12,000 rpm for 10 min, and the supernatants were collected. Protein concentrations were determined using a BCA protein assay kit with bovine serum albumin (BSA) at 5 mg/mL as the standard. After denaturation, 20 μg of protein from each sample was loaded onto a 10% sodium dodecyl sulfate‐polyacrylamide gel (SDS‐PAGE) and separated at 120 V. The proteins were subsequently transferred onto PVDF membranes at 400 mA for 60 min. The membranes were blocked with protein‐free Western blocking buffer for 30 min at room temperature and then washed with TBST.

The membranes were incubated overnight at 4°C with gentle shaking using primary antibodies against iNOS (1:2000), Arg‐1 (1:2000), phospho‐NF‐κB p65 (Ser468) (1:1000), NF‐κB p65 (1:1000), phospho‐p38 MAPK (Thr180/Tyr182) (1:1000), p38 MAPK (1:2000), and β‐tubulin (1:3000), all purchased from Proteintech (China). After washing with TBST, the membranes were incubated with the corresponding HRP‐conjugated secondary antibodies (1:3000; Beyotime, China) for 1 h. Protein bands were visualized using an enhanced chemiluminescence (ECL) detection system. The band intensities were quantified using Image J software. The expression levels of target proteins were normalized to the corresponding loading control and expressed relative to the control group.

### 2.12 Molecular Docking Analysis

The three‐dimensional structure of the extracellular domain of TLR2/4 was obtained from the RCSB database (https://www.rcsb.org). Based on the repeating unit structure of IBPS‐3A deduced in Section 3.4, the three‐dimensional simulated structures of backbone and side‐chain repeating fragments were generated on the GLYCAM‐Web server (https://glycam.org/).

Selected pocket residues of TLR2/4 were defined as flexible side chains. The ligand was energy‐minimized prior to docking. Semi‐flexible docking was performed using AutoDock Vina with exhaustiveness = 24. Up to nine binding modes were generated. The docking conformation with the lowest binding free energy was selected for interaction analysis. Visualization analysis of the interaction complex was carried out using Discovery Studio software.

### 2.13 Graphic Processing and Statistical Analysis

All cell‐based experiments were performed using three independent biological replicates, with more than three technical replicates (parallel wells) included for each treatment group. Graphs were plotted using Origin 2021 software. Data are presented as the mean ± standard deviation (SD). Statistical significance was evaluated by one‐way analysis of variance (ANOVA) using SPSS 25 software, with *p* < 0.05 (*), *p* < 0.01 (**), and *p* < 0.001 (***) considered statistically significant.

## 3 Results

### 3.1 Isolation and Purification of Polysaccharides From Sweet Potato Vines

Extraction flowchart of IBPS‐3A is shown in Figure 1A. A yield of 1.26 g of crude polysaccharide IBPSs was obtained from 100 g of sweet potato vines. This crude extract was subsequently processed via ion‐exchange chromatography for further purification, producing three fractions, namely the anticipated neutral fraction IBPS‐1, and two potentially acidic polysaccharide fractions, IBPS‐2 and IBPS‐3 (Figure 1B). Preliminary experiments showed that IBPS‐3 at a low concentration of 12.5 μg/mL significantly promoted the polarization of RAW264.7 cells compared with IBPS‐1 and IBPS‐2 (Figure S1). Consequently, IBPS‐3 was subsequently fractionated using a Superdex G‐200 to obtain a single major fraction, designated as IBPS‐3A (Figure 1C), and 280 mg of purified IBPS‐3A was obtained from 100 g of dried sweet potato vines, giving a final extraction yield of 0.28%.

Chemical analysis showed that IBPS‐3A contained 96.23% ± 1.3% total sugar, with no detectable protein contamination. Using the endotoxin assay kit, the endotoxin content of IBPS‐3A was determined to be 0.000125–0.00025 EU/μg, indicating a low level of residual endotoxin in IBPS‐3A (Tian et al. 2025). In the HPGPC analysis, IBPS‐3A exhibited a sharp, symmetric peak, reflecting a uniform molecular weight profile with good purity (Figure 1D). Based on the elution time, the weight‐average Mw of IBPS‐3A was calculated to be 56,077 Da (Mn = 29,711 Da, Mz = 87,923 Da, PDI = 1.88). The UV–Vis spectrum of IBPS‐3A showed a characteristic polysaccharide absorption between 200 and 400 nm, and no notable absorbance peaks corresponding to proteins or nucleic acids were observed (Figure 1E).

### 3.2 FT‐IR and Monosaccharide Composition Analysis

FT‐IR spectroscopic analysis revealed characteristic absorption peaks of the polysaccharides in IBPS‐3A. A strong, broad absorption peak at 3428.77 cm^−1^ was observed, attributed to the O‐H stretching vibration due to intermolecular and intramolecular hydrogen bonds (Zhao et al. 2019). The signal at 1612.66 cm^−1^ corresponded to the asymmetric stretching vibration of the free carboxylate group (COO‐) in uronic acids, indicating that IBPS‐3A is an acidic polysaccharide (Han et al. 2024). Peaks at 1100.38 cm^−1^ and 1016.71 cm^−1^ arose from the bending vibrations of C‐O bonds in C‐O‐H or C‐O‐C structures, confirming the presence of pyranose rings (Pason et al. 2023). An absorption at 893.86 cm^−1^, characteristic of the β‐configuration in pyranosidic linkages, suggested the presence of β‐type pyranose sugars in IBPS‐3A (Figure 1F) (Chen et al. 2023).

The monosaccharide composition of IBPS‐3A was analyzed using a Thermo U3000 liquid chromatography system. IBPS‐3A was identified as an acidic heteropolysaccharide, primarily composed of galacturonic acid (GalA), galactose (Gal), arabinose (Ara), rhamnose (Rha), and trace amounts of glucose (Glc) (Figure 1G, Table S1). GalA and Gal collectively accounted for approximately 80% of the total molar percentage, displaying the typical characteristics of pectic polysaccharides (Caffall and Mohnen 2009). This suggests that IBPS‐3A could be classified as a pectic polysaccharide.

SEM was performed to observe the surface morphology of the IBPS‐3A sample. At both low and high magnifications, the sample exhibited a uniformly distributed, sheet‐like structure with a smooth surface and a closely packed arrangement (Figure 1H).

### 3.3 Methylation Analysis

Methylation analysis is a standard method used to elucidate the types of glycosidic linkages between sugar residues (Yang et al. 2022). Following uronic acid reduction, IBPS‐3A was methylated and the resulting partially methylated alditol acetates (PMAAs) were examined by gas chromatography–mass spectrometry (GC–MS). As shown in the corresponding figure (Figure S2), ten PMAAs were identified in the total ion chromatogram. These peaks were characterized as t‐Ara*f*, 1,5‐Ara*f*, t‐Gal*p*, t‐Gal*p*A, 1,2,4‐Rha*p*, 1,4‐Gal*p*, 1,4‐Gal*p*A, 1,4‐Glc*p*, 1,4‐Glc*p*A, and 1,3‐Gal*p*. The detailed results are presented in Table 1 and Figure 2.

**TABLE 1:** Methylation analysis and deduced glycosidic linkages of the constituent sugars of IBPS‐3A.

Type of linkage	Methylated sugar	Molar ratio (%)	RT (min)	Mass fragments (m/z)
t‐Ara*f*	1,4‐di‐*O*‐acetyl‐2,3,5‐tri‐*O*‐methyl arabinitol	1.8	11.733	71,87,102,118,161,162
1,5‐Ara*f*	1,4,5‐tri‐*O*‐acetyl‐2,3‐di‐*O*‐methyl arabinitol	2.6	15.479	71,87,102,118,129,162,189
t‐Gal*p*	1,5‐di‐*O*‐acetyl‐2,3,4,6‐tetra‐*O*‐methyl galactitol	3.0	17.512	59,73,88,102,118,129,145,161,205
t‐Gal*p*A	1,5‐di‐*O*‐acetyl‐2,3,4,6‐tetra‐*O*‐methyl galactitol	4.7	17.512	59,73,88,102,118,131,147,162,163,207
1,2,4‐Rha*p*	1,2,4,5‐tetra‐*O*‐acetyl‐6‐deoxy‐3‐*O*‐methyl rhamnitol	3.6	18.438	43,88,101,130,143,190,203
1,4‐Gal*p*	1,4,5‐tri‐*O*‐acetyl‐2,3,6‐tri‐*O*‐methyl galactitol	4.2	19.983	43,87,102,118,129,144,162,173,233
1,4‐Gal*p*A	1,4,5‐tri‐*O*‐acetyl‐2,3,6‐tri‐*O*‐methyl galactitol	76	19.983	43,87,102,118,129,144,162,175,235
1,4‐Glc*p*	1,4,5‐tri‐*O*‐acetyl‐2,3,6‐tri‐*O*‐methyl glucitol	1.3	20.115	43,71,87,102,113,118,129,145,162,173,233
1,4‐Glc*p*A	1,4,5‐tri‐*O*‐acetyl‐2,3,6‐tri‐*O*‐methyl glucitol	1.2	20.115	43,71,87,102,113,118,129,147,162,175,233,235
1,3‐Gal*p*	1,3,5‐tri‐*O*‐acetyl‐2,4,6‐tri‐*O*‐methyl galactitol	1.6	20.384	59,87,101,118,129,161,234,277

**FIGURE 2:**
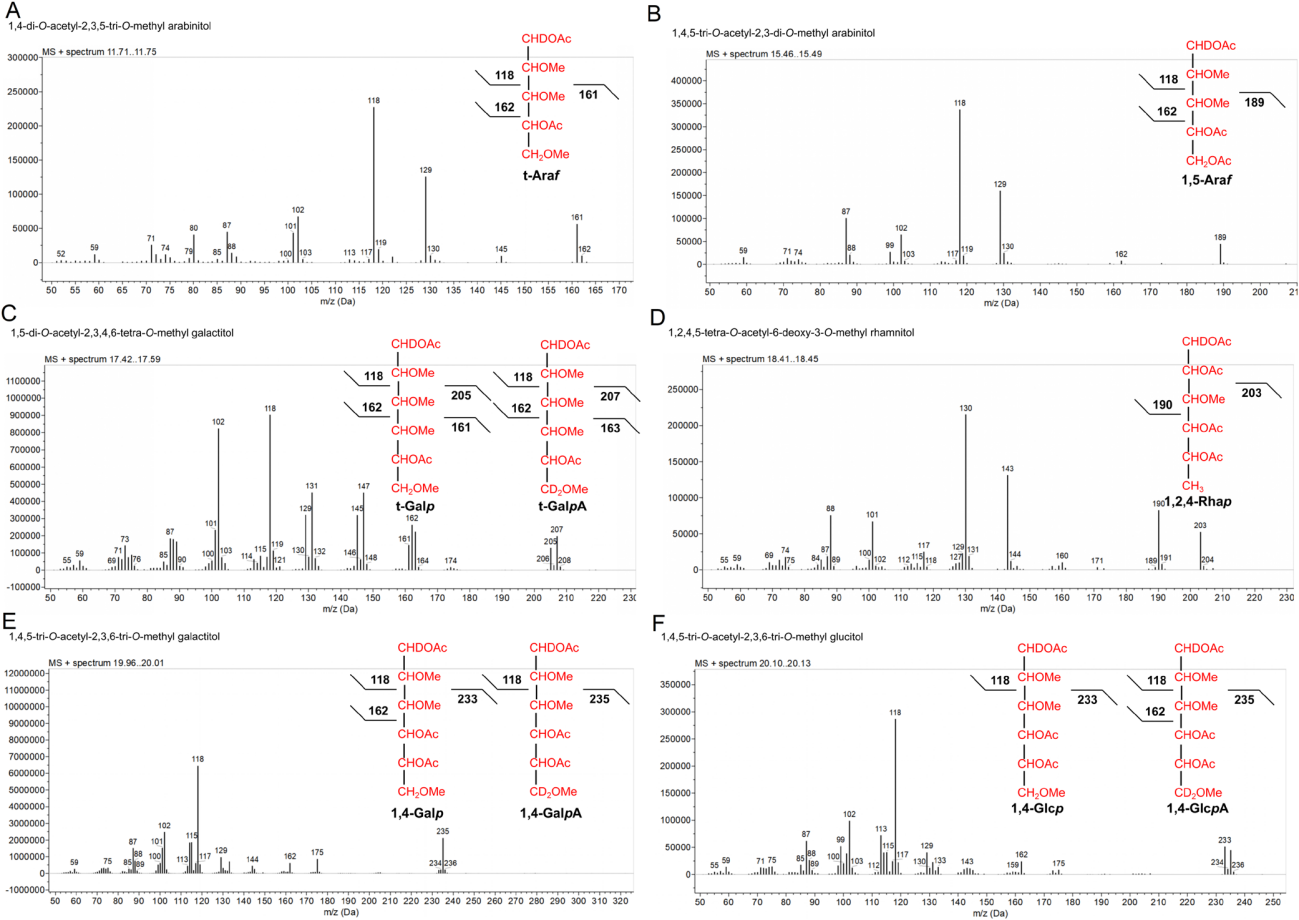
Methylation analysis of IBPS‐3A. (A) EI‐MS spectrum of 1,4‐di‐*O*‐acetyl‐2,3,5‐tri‐*O*‐methyl arabinitol; (B) EI‐MS spectrum of 1,4,5‐tri‐*O*‐acetyl‐2,3‐di‐*O*‐methyl arabinitol; (C) EI‐MS spectrum of 1,5‐di‐*O*‐acetyl‐2,3,4,6‐tetra‐*O*‐methyl galactitol; (D) EI‐MS spectrum of 1,2,4,5‐tetra‐*O*‐acetyl‐6‐deoxy‐3‐*O*‐methyl rhamnitol; (E) EI‐MS spectrum of 1,4,5‐tri‐*O*‐acetyl‐2,3,6‐tri‐*O*‐methyl galactitol; (F) EI‐MS spectrum of 1,4,5‐tri‐*O*‐acetyl‐2,3,6‐tri‐*O*‐methyl glucitol.

Among these, 1,4‐Gal*p*A accounted for over 70% of the total, suggesting that the backbone of IBPS‐3A is predominantly composed of the HG domain. The presence of 1,2,4‐Rhap indicates the presence of the RG‐I domain structure. Linkages such as 1,5‐Ara*f*, 1,3‐Gal*p*, and 1,4‐Gal*p* imply the existence of arabinogalactan side chains.

### 3.4 NMR Spectroscopic Analysis

To further resolve the structural attributes of the polysaccharide sample IBPS‐3A, one‐dimensional NMR spectra, including ^1^H‐NMR, ^13^C‐NMR, and DEPT‐135, along with two‐dimensional NMR spectra, namely COSY, HSQC, TOCSY, HMBC, and NOESY, were acquired. The corresponding spectra are shown in the figures (Figure 3; Figure S3).

**FIGURE 3:**
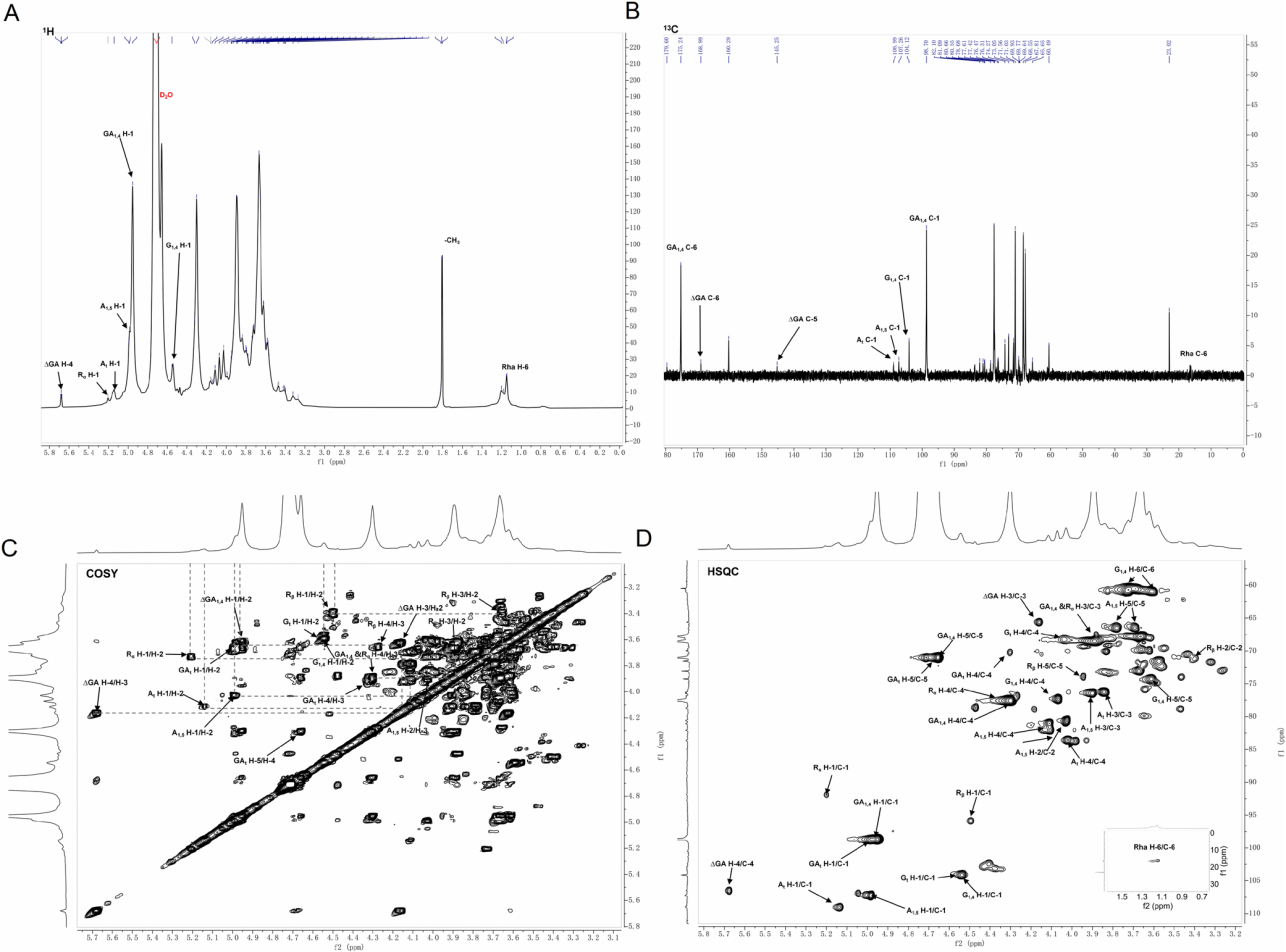
Nuclear magnetic resonance spectroscopy of IBPS‐3A. (A) ^1^H NMR spectrum of IBPS‐3A; (B) ^13^C NMR spectrum of IBPS‐3A; (C) COSY NMR spectrum of IBPS‐3A; (D) HSQC NMR spectrum of IBPS‐3A.

^1^H NMR and ^13^C NMR spectrum of IBPS‐3A are shown in (Figure 3A,B). In the ^1^H‐NMR spectrum of IBPS‐3A, abundant proton resonance signals appeared in the region of δ 3.0–5.5 ppm, exhibiting significant overlap. In the anomeric region (δ 4.3–5.5 ppm), multiple anomeric proton signals were observed, indicating the presence of various sugar residues. Resonance signals in the high‐field region of δ 1.10–1.30 ppm correspond to the characteristic methyl protons (H‐6) of rhamnose (Rha) residues (Ding et al. 2015; Liu, Yi, et al. 2025; Shakhmatov and Makarova 2025). A cross‐peak at δ 1.19/16.54 ppm was identified in the HSQC spectrum (Figure 3D). The low intensity of these signals in both the ^1^H and ^13^C spectra suggests the presence of only a limited quantity of rhamnose residues, aligning with the monosaccharide composition analysis.

Based on the chemical shifts identified in these anomeric regions, and through comprehensive analysis of COSY, HSQC, HMBC (Figures 3C,D and 4A), TOCSY and NOESY (Figure 4B,C) spectra in conjunction with ^1^H‐NMR, ^13^C‐NMR (Figure 3B), and DEPT‐135 (Figure S3) data, along with monosaccharide composition and methylation analysis results, the sugar residues were assigned by referencing published chemical shift data for similarly substituted residues (Košťálová and Hromádková 2019; Smith et al. 2020; Chen et al. 2021; Tang et al. 2021; Peng et al. 2023). The major sugar residue types were sequentially labeled as: GA_1,4_, R_α_, R_β_, GA_t_, G_1,4_, G_t_, A_t_, A_1,5_, ΔGA, and Rha_1,2,4_. The ^1^H and ^13^C chemical shift assignments for the main types of sugar residues in the polysaccharide sample are summarized in Table 2.

**FIGURE 4:**
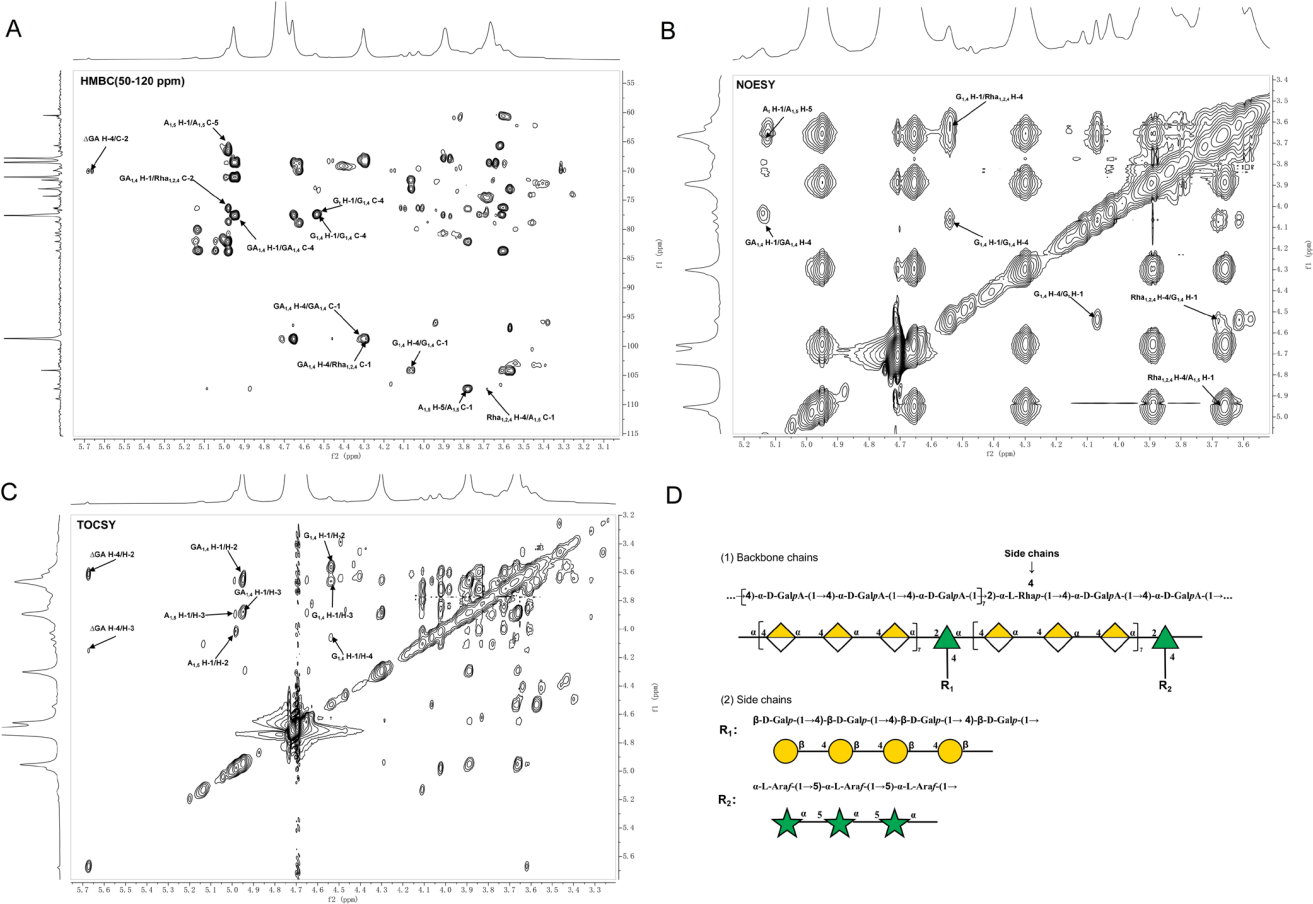
Nuclear magnetic resonance spectroscopy of IBPS‐3A. (A) HMBC NMR spectrum of IBPS‐3A; (B) NOESY NMR spectrum of IBPS‐3A; (C) TOCSY NMR spectrum of IBPS‐3A; (D) The proposed structural diagram of IBPS‐3A.

**TABLE 2:** Chemical shifts of assignments in the ^1^H and ^13^C NMR spectra of IBPS‐3A.

Glycosyl residues		Chemical shift δ(ppm)						
			1	2	3	4	5	6a/6b
GA_1,4_	→4)‐α‐D‐Gal*p*A‐(1→	H	4.95	3.65	3.89	4.29	4.65	
		C	98.70	67.76	68.43	77.61	71.04	175.22
R_α_	→4)‐ α‐D‐Gal*p*A	H	5.19	3.72	3.88	4.29	4.64	
		C	91.95	67.76	68.57	77.51	70.94	175.22
R_β_	→4)‐β‐D‐Gal*p*A	H	4.49	3.39	3.64	4.26	3.94	
		C	95.86	71.22	72.07	76.79	74.01	174.29
GA_t_	α‐D‐GalpA‐(1→	H	4.95	3.66	3.88	4.30	4.64	
		C	98.79	67.87	68.33	70.24	71.15	175.22
G_1,4_	→4)‐β‐D‐Gal*p*‐(1→	H	4.53	3.58	3.67	4.06	3.62	3.61/3.72
		C	104.08	71.61	73.09	77.40	74.33	60.73
G_t_	β‐D‐Gal*p*‐(1→	H	4.53	3.57	3.67	4.04	3.60	3.61/3.71
		C	104.02	71.72	73.20	68.22	74.43	60.87
A_t_	α‐L‐Ara*f*‐(1→	H	5.13	4.11	3.83	4.02	3.60/3.72	
		C	109.10	81.14	76.48	83.61	60.98	
A_1,5_	→5)‐α‐L‐Ara*f*‐(1→	H	4.98	4.02	3.90	4.10	3.69/3.78	
		C	107.06	80.61	76.50	82.07	66.52	
ΔGA	α‐D‐Δ4,5‐GalpA‐(1→	H	4.98	3.62	4.15	5.67	—	
		C	98.9	69.91	65.64	106.52	145.17	169.06
Rha_1,2,4_	→2,4)‐α‐L‐Rha*p*‐(1→	H	5.15	4.08	3.83	3.67	3.93	1.19
		C	98.23	77.40	68.43	79.76	68.68	16.54

Sequential linkages between sugar residues were further elucidated using long‐range correlations observed in the HMBC (Figure 4A) and NOESY (Figure 4B) spectra. The subsequent coupling signals were assigned as follows: (1) In the HMBC spectrum, a correlation peak between the H‐1 (δ 4.95 ppm) of residue GA_1,4_ and the C‐4 (δ 77.61 ppm) of another GA_1,4_ residue (GA_1,4_ H‐1/GA_1,4_ C‐4), along with a correlation between the H‐4 (δ 4.29 ppm) of GA_1,4_ and the C‐1 (δ 98.70 ppm) of a GA_1,4_ residue (GA_1,4_ H‐4/GA_1,4_ C‐1), together with a cross‐peak between GA_1,4_ H‐1 and GA_1,4_ H‐4 in the NOESY spectrum, indicate a →4)‐α‐D‐Gal*p*A‐(1→4)‐α‐D‐Gal*p*A‐(1→ linkage. (2) HMBC correlations between GA_1,4_ H‐1 and the C‐2 (δ 77.40 ppm) of residue Rha_1,2,4_ (GA_1,4_ H‐1/Rha_1,2,4_ C‐2), and between GA_1,4_ H‐4 and the C‐1 (δ 98.23 ppm) of Rha_1,2,4_ (GA_1,4_ H‐4/Rha_1,2,4_ C‐1), indicate a →4)‐α‐D‐Gal*p*A‐(1→2,4)‐α‐L‐Rha*p*‐(1→ linkage, with a connection at the *O*‐2 position. (3) The HMBC correlations between G_1,4_ H‐1 (δ 4.53 ppm) and G_1,4_ C‐4 (δ 77.40 ppm) (G_1,4_ H‐1/G_1,4_ C‐4), and between G_1,4_ H‐4 (δ 4.06 ppm) and G_1,4_ C‐1 (δ 104.08 ppm) (G1,4 H‐4/G1,4 C‐1), along with a G_1,4_ H‐1/G_1,4_ H‐4 cross‐peak in NOESY, confirm a →4)‐β‐D‐Gal*p*‐(1→4)‐β‐D‐Gal*p*‐(1→ linkage. (4) An HMBC correlation between G_t_ H‐1 (δ 4.53 ppm) and G_1,4_ C‐4 (δ 77.40 ppm) (G_t_ H‐1/G_1,4_ C‐4), and a NOESY cross‐peak between G_1,4_ H‐4 (δ 4.06 ppm) and G_t_ H‐1 (δ 4.06 ppm) (G_1,4_ H‐4/G_t_ H‐1) confirm a β‐D‐Gal*p*‐(1 → 4)‐β‐D‐Gal*p*‐(1 → linkage. (5) An HMBC correlation between A_1,5_ H‐1 (δ 4.98 ppm) and its own C‐5 (δ 66.52 ppm) (A_1,5_ H‐1/A_1,5_ C‐5) indicates a →5)‐α‐L‐Ara*f*‐(1→5)‐α‐L‐Ara*f*‐(1→ linkage at the *O*‐5 position. (6) A NOESY cross‐peak between A_t_ H‐1 (δ 5.13 ppm) and A_1,5_ H‐5 (δ 3.69 ppm) (A_t_ H‐1/A_1,5_ H‐5) suggests an additional connection for this α‐L‐Ara*f*‐(1 → 5)‐α‐L‐Araf‐(1 → linkage. (7) An HMBC correlation between Rha_1,2,4_ H‐4 (δ 3.67 ppm) and A_1,5_ C‐1 (δ 107.06 ppm) (Rha_1,2,4_ H‐4/A_1,5_ C‐1) and the NOESY cross‐peak between Rha_1,2,4_ H‐4 (δ 3.67 ppm) and A_1,5_ H‐1 (δ 4.98 ppm) (Rha_1,2,4_ H‐4/A_1,5_ H‐1) indicates a →5)‐α‐L‐Ara*f*‐(1→2,4)‐α‐L‐Rha*p*‐(1→ linkage at the *O*‐4 position. (8) The NOESY cross‐peak between G_1,4_ H‐1 (δ 4.53 ppm) and Rha_1,2,4_ H‐4 (δ 3.67 ppm) (G_1,4_ H‐1/Rha_1,2,4_ H‐4) suggests a →4)‐β‐D‐Gal*p*‐(1→2,4)‐α‐L‐Rha*p*‐(1→ linkage at the *O*‐4 position.

Based on the basis of a comprehensive evaluation of the monosaccharide composition and methylation results along with the 1D and 2D NMR data for IBPS‐3A, the polysaccharide sample was found to be a complex heteropolysaccharide. To a large extent, it can be inferred that the structure is primarily composed of extensive HG domains and partially branched rhamnogalacturonan I (RG‐I) domains, forming a pectic polysaccharide. The proposed schematic structural elements are shown in Figure 4D.

### 3.5 Induction of M1 Polarization in RAW264.7 Cells by IBPS‐3A

Upon treatment with IBPS‐3A, a greater proportion of RAW264.7 cells adopted a spindle‐shaped morphology with elongated pseudopodia, characteristic of M1‐polarized cells (Figure 5A). Furthermore, we observed a significant increase in cell viability in RAW264.7 cells exposed to IBPS‐3A. CCK‐8 assay confirmed that IBPS‐3A significantly enhanced cell proliferation in a concentration‐dependent manner (Figure 5B). To further verify that IBPS‐3A induces M1 polarization in RAW264.7 cells, a series of M1‐related markers were examined. The expression of the M1‐associated marker iNOS increased, whereas the M2‐related marker Arg‐1 level decreased (Figure S4). The concentrations of relevant secreted factors in the cell culture supernatant were measured by ELISA. After 24 h of treatment with IBPS‐3A, the secretion of TNF‐α, IL‐6, and NO increased in a concentration‐dependent fashion (Figure 5C–E). Within the concentration range tested, IBPS‐3A exhibited a more pronounced promoting effect compared with LPS; that is, promoting macrophage M1 polarization and stimulating the release of pro‐inflammatory mediators including TNF‐α, IL‐6, and NO. Immunofluorescence staining also provided a more direct visual confirmation of the increased intracellular iNOS levels (Figure 5F).

**FIGURE 5:**
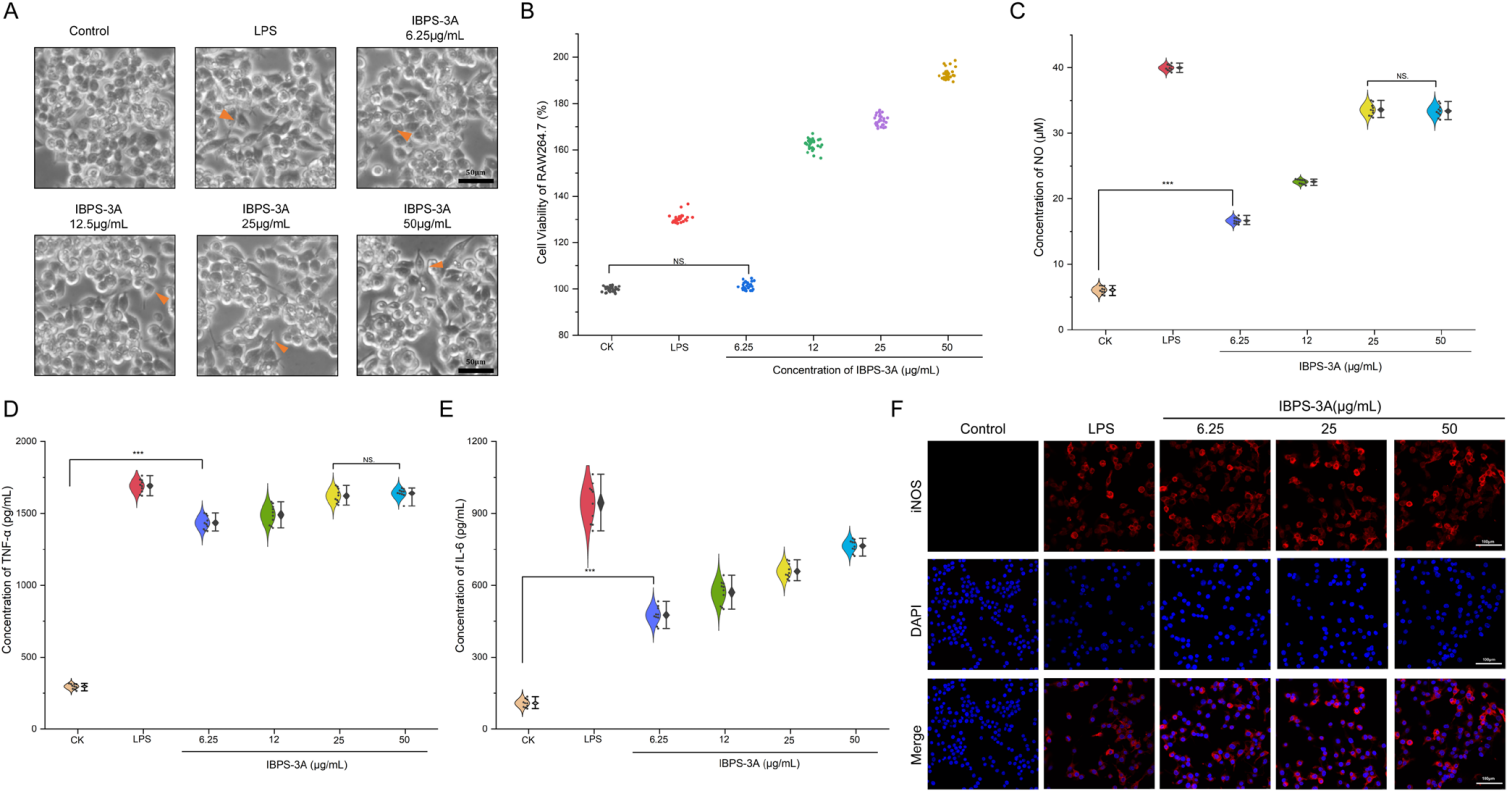
Induction of M1 polarization in RAW264.7 cells by IBPS‐3A. (A) Morphological characteristics of RAW264.7 cells after treatment with IBPS‐3A; (B) Effect of IBPS‐3A on the viability of RAW264.7 cells; (C) Nitric oxide (NO) production in the supernatant of RAW264.7 cells treated with IBPS‐3A; (D) TNF‐α level in the supernatant of RAW264.7 cells treated with IBPS‐3A; (E) IL‐6 level in the supernatant of RAW264.7 cells treated with IBPS‐3A; (F) Immunofluorescence staining of intracellular iNOS. ***p < 0.001.

### 3.6 Cytotoxic Effect of Conditioned Medium From IBPS‐3A‐Treated RAW264.7 Cells on B16 Cells

B16 cells were treated directly with IBPS‐3A at varying concentrations (6.25, 12.5, 25, 50, 200 μg/mL), and cell viability was assessed after 24 h. IBPS‐3A had no obvious direct effect on the growth or proliferation of B16 cells (Figure 6A,B). To investigate whether IBPS‐3A exerts cytotoxic effects on B16 cells via its influence on RAW264.7 cell secretion, conditioned medium (CM) collected from RAW264.7 cells treated with IBPS‐3A was used to assess B16 cell growth (Ni et al. 2019). As shown in Figure S5, at CM concentrations of 1.25% and 2.5%, none of the treatment groups' conditioned medium exhibited cytotoxic effects on the B16 cells. When the CM concentration was increased to 5%, the CM from the highest tested dose of IBPS‐3A reduced B16 cell viability by 13.7%. At a 10% CM concentration, the highest IBPS‐3A dose reduced cell viability by 16.5% (Figure 6C).

**FIGURE 6:**
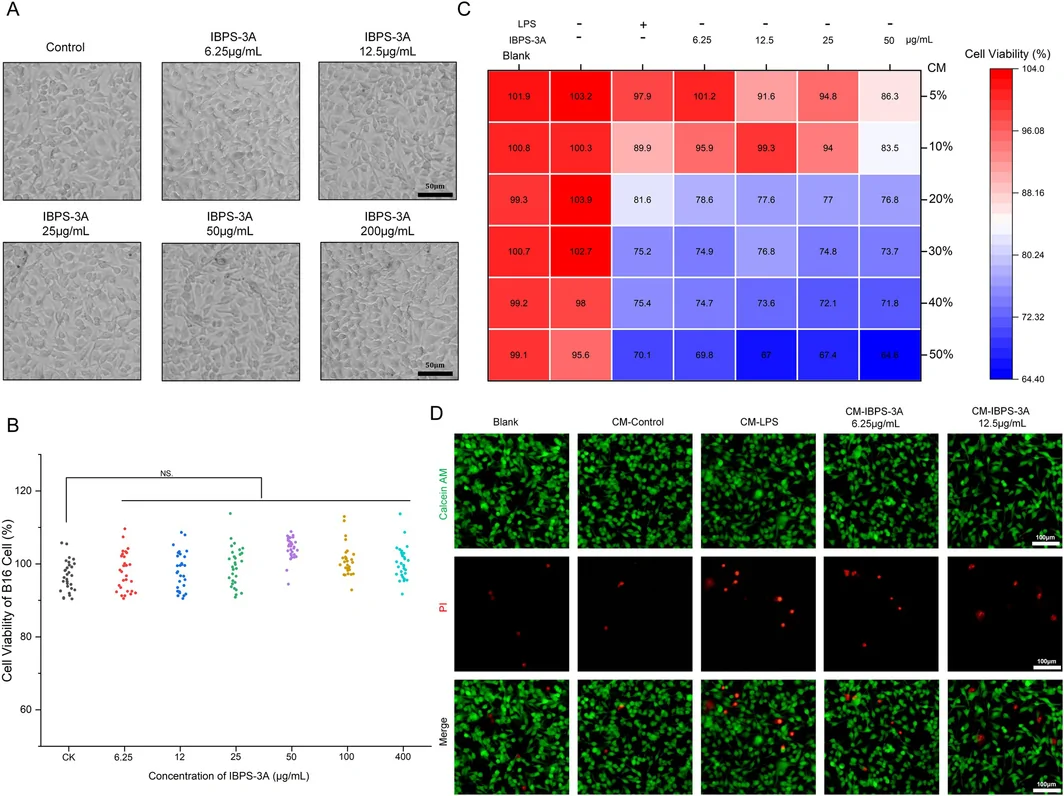
The cytotoxic effect of conditioned medium on B16 cells. (A) IBPS‐3A showed no obvious direct effect on the growth of B16 cells; (B) Effect of IBPS‐3A on B16 cell viability; (C) The cytotoxic effect of different ratios of conditioned medium on B16 cells; (D) PI staining of B16 cells after treatment with conditioned medium, Calcein‐AM (green, live cells) and PI (red, dead cells).

Upon further increasing the CM concentration to 20% or higher, CM from RAW264.7 cells treated with either LPS or varying concentrations of IBPS‐3A significantly reduced B16 cell viability. In contrast, the CM from untreated RAW264.7 cells showed no cytotoxic effects (Figure 6C). Compared with the untreated control, cell viability across the different treatment groups was reduced by approximately 30%. At the highest tested IBPS‐3A dose with a 50% proportion of conditioned medium, B16 cell viability was reduced by 35%, indicating that the cytotoxic effect of IBPS‐3A under these experimental conditions was approaching saturation (Figure 6C). Furthermore, propidium iodide (PI) staining visually demonstrated that treatment with IBPS‐3A‐conditioned medium markedly decreased the density of living cells and caused a rise in dead cell counts (Figure 6D).

### 3.7 Cytotoxic Effect of IBPS‐3A on B16 Cells in a Co‐Culture System

To demonstrate the cytotoxic effect of IBPS‐3A on B16 cells resulting from the induction of RAW264.7 polarization and cytokine secretion, a Transwell‐based co‐culture system was established with B16 and RAW264.7 cells, which were treated with varying concentrations of IBPS‐3A. At a low B16 cell density (5 × 10^4^ cells per well), IBPS‐3A enhanced the cytotoxic effect mediated by RAW264.7 cells from 10.2% to a maximum of 20.1% (Figure S6). At a higher B16 cell density (1.5 × 10^5^ cells per well), the reduction in B16 cell viability increased from 11.2% to a maximum of 29.5% (Figure 7A–C). Furthermore, this cytotoxic effect was amplified with an increase in RAW264.7 cell density. When the RAW264.7 cell density was raised to 8 × 10^5^ cells per well and treated with 50 μg/mL IBPS‐3A, the cytotoxic effect was enhanced by 25.8% compared to the untreated control (Figure 7C).

**FIGURE 7:**
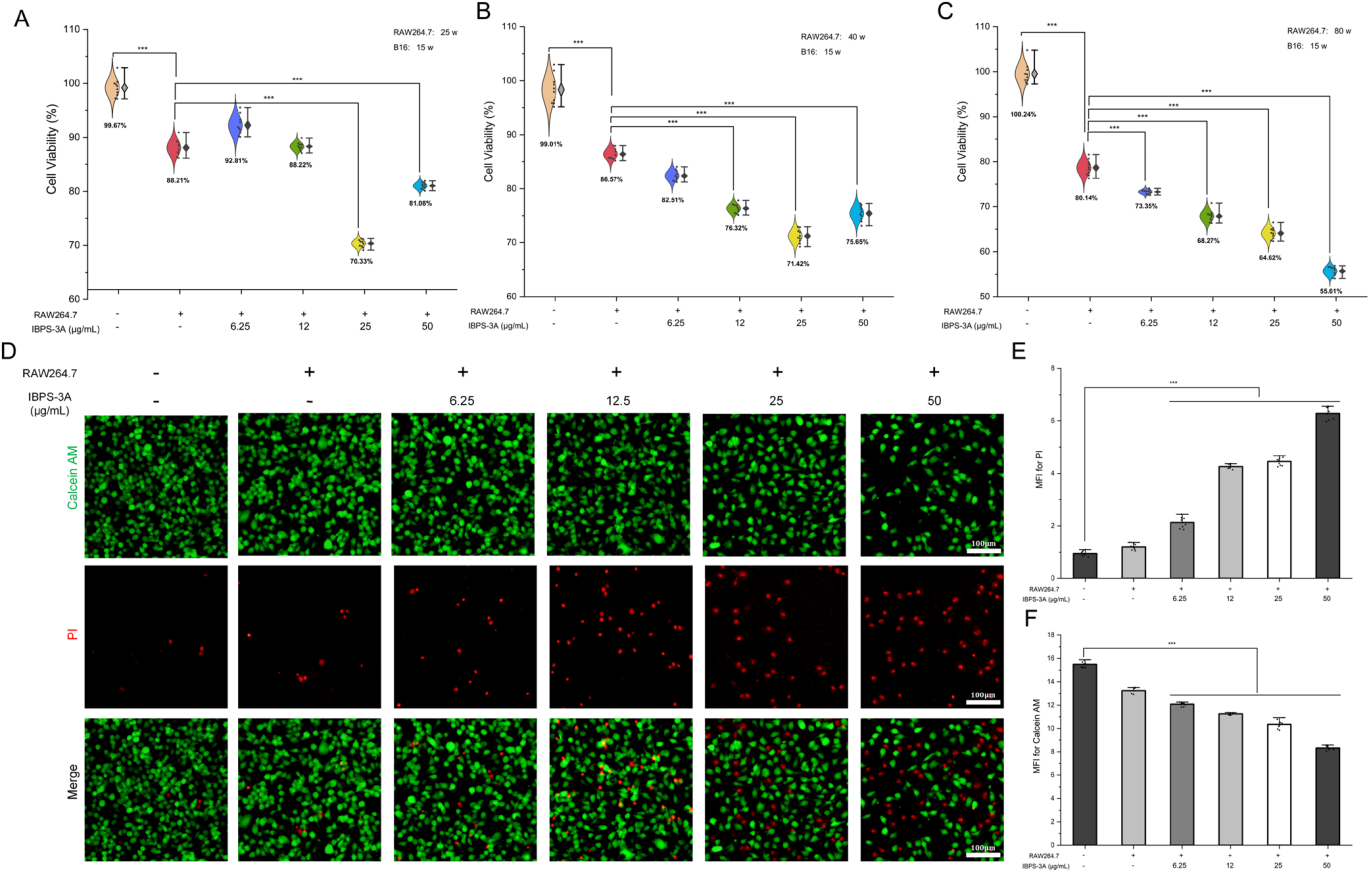
Cytotoxic effect of IBPS‐3A on B16 cells in a co‐culture system. (A–C) The cytotoxic effect of IBPS‐3A on B16 cells in a co‐culture system; (D) PI staining of B16 cells after co‐culture, Calcein‐AM (green, live cells) and PI (red, dead cells); (E) The mean fluorescence intensity (MFI) of PI represents to the number of dead cells; (F) The mean fluorescence intensity (MFI) of Calcein AM represents to the number of live cells. ***p < 0.001.

Live/dead staining of B16 cells in the co‐culture system revealed that the introduction of IBPS‐3A notably lowered the density of live cells and increased that of dead cells (Figure 7D–F). At IBPS‐3A concentrations below 50 μg/mL, the decrease in live cell number and the increase in dead cell number both exhibited a concentration‐dependent trend. The strongest cytotoxic response was detected at 50 μg/mL IBPS‐3A, where the density of live cells was reduced to less than half of that in the control group without co‐culture treatment, which is consistent with the CCK‐8 assay results. In conclusion, the M1 polarization of RAW264.7 cells induced by IBPS‐3A can effectively and substantially reduce B16 cell viability in a co‐culture system.

### 3.8 IBPS‐3A Activates RAW264.7 Cell M1 Polarization via TLR2 and TLR4

To investigate the primary receptors through which IBPS‐3A acts on RAW264.7 cells, the TLR2 inhibitor C29 and the TLR4 inhibitor TAK‐242 were employed (Qin et al. 2022). Initially, the safety of C29 and TAK‐242 in RAW264.7 cells and their inhibitory effects on LPS‐induced M1 polarization were assessed. Concentrations of C29 below 50 μmol/L and TAK‐242 below 10 μmol/L did not affect RAW264.7 cell viability (Figure 8A,C). C29 showed a certain suppressive effect on LPS‐stimulated TNF‐α secretion, with little difference observed across its various concentrations, suggesting that the TLR2 receptor plays a supplementary role in recognizing LPS signaling (Figure 8B). In contrast, TAK‐242 substantially abrogated the LPS‐stimulated rise of TNF‐α secretion; at 10 μmol/L, TNF‐α levels were even lower than those in the control group, underscoring the important role of the TLR4 receptor in LPS‐induced M1 polarization (Figure 8D). C29 and TAK‐242 were used at concentrations of 50 and 10 μmol/L, respectively, in subsequent experiments.

**FIGURE 8:**
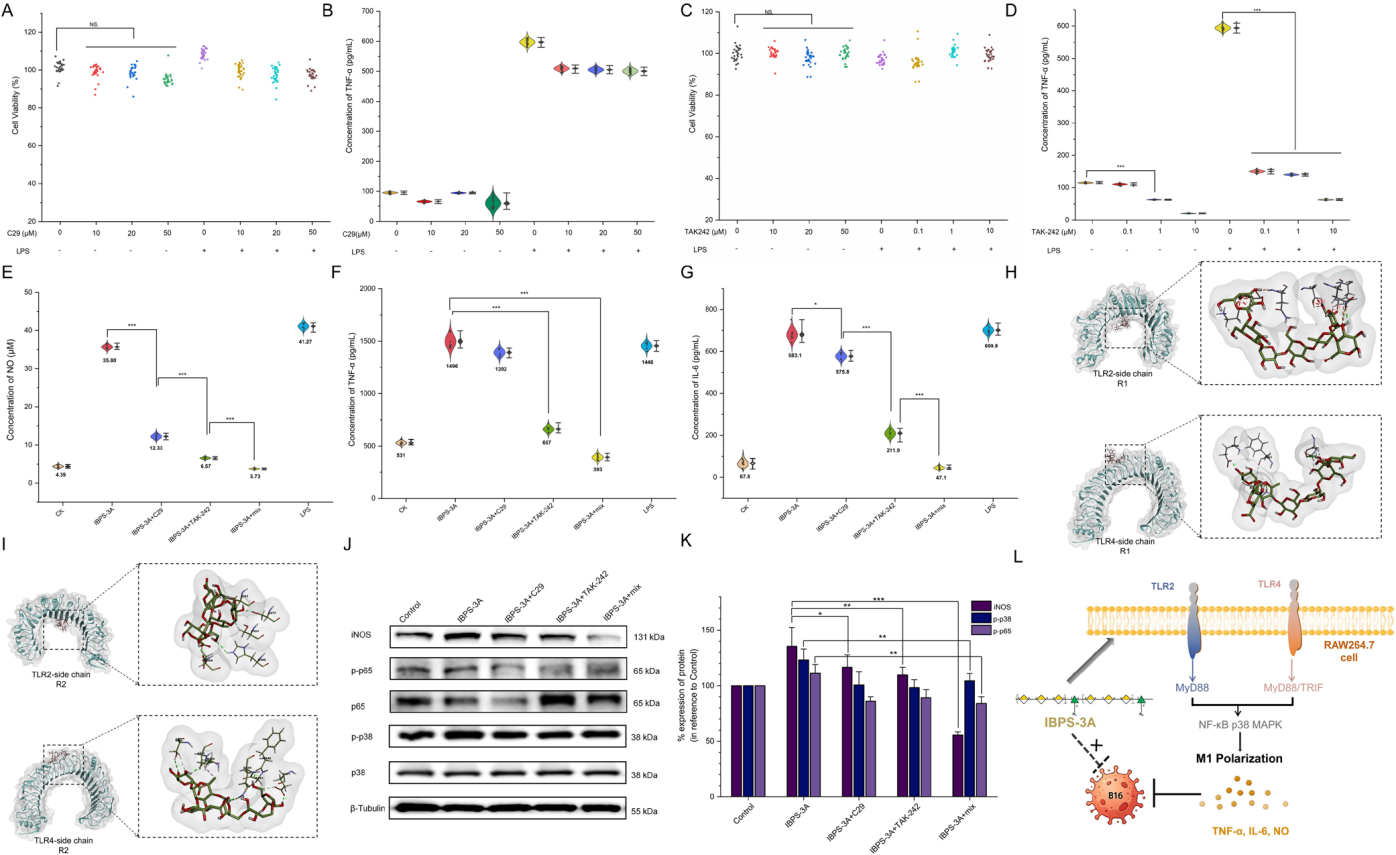
IBPS‐3A activates RAW264.7 cell M1 polarization via TLR2 and TLR4. (A) Effect of C29 on RAW264.7 macrophage viability; (B) Effect of C29 on TNF‐α secretion by RAW264.7 macrophages; (C) Effect of TAK‐242 on RAW264.7 macrophage viability; (D) Effect of TAK‐242 on TNF‐α secretion by RAW264.7 macrophages; (E) Effects of inhibitors on IBPS‐3A‐induced NO production; (F) Effects of inhibitors on IBPS‐3A‐induced TNF‐α production; (G) Effects of inhibitors on IBPS‐3A‐induced IL‐6 production; (H) Molecular docking simulation diagrams of side chain R1 with TLR2/4; (I) Molecular docking simulation diagrams of side chain R2 with TLR2/4. (J) Expression of proteins in the NF‐κB and MAPK signaling pathways detected by Western blotting; (K) Quantitative analysis of relative protein expression levels determined by densitometric quantification of Western blot bands. (L) The mechanism by which IBPS‐3A induced M1 polarization of macrophages via activation of the Toll‐like receptor 2/4 signaling pathway. *p < 0.05, **p < 0.01 and ***p < 0.001, respectively.

Both inhibitors significantly suppressed the IBPS‐3A‐induced increase in NO secretion (Figure 8E). C29 slightly reduced the secretion of TNF‐α and IL‐6 stimulated by IBPS‐3A, whereas TAK‐242 exhibited a more pronounced inhibitory effect (Figure 8F,G). When co‐treated with both inhibitors and IBPS‐3A, the levels of all three inflammatory factors were lower than those in the blank control (CON) group, suggesting that TLR2 and TLR4 are the potential receptors mediating IBPS‐3A‐induced M1 polarization (Figure 8E–G). In addition, we modeled the main chain and side chains of IBPS‐3A using the Glycam web server (https://glycam.org/). Molecular docking calculations were performed using AutoDock Vina for the TLR2‐main chain, TLR4‐main chain, TLR2‐side chains, and TLR4‐side chains. Based on theoretical calculations, IBPS‐3A is predicted to have potential binding interactions with TLR2 and TLR4. Moreover, the binding energies of TLR2/4 with the IBPS‐3A side chains were significantly higher (TLR2‐side chain R1: −5.02 kcal/mol; TLR4‐side chain R1: −5.09 kcal/mol; TLR4‐side chain R2: −5.48 kcal/mol) than those with the main chain (TLR2‐main chain: −3.27 kcal/mol and TLR4‐main chain: −4.22 kcal/mol) (Figure 8H,I; Figure S7).

Furthermore, Western blotting was performed to assess protein expression levels in the NF‐κB and MAPK pathways after 24 h of treatment with IBPS‐3A, C29, and TAK‐242. The findings revealed that the ratios of p‐p65/p65 and p‐p38/p38 increased following IBPS‐3A treatment but decreased upon co‐treatment with the inhibitors (Figure 8J,K), demonstrating the involvement of the TLR2/4‐MyD88/TRIF‐NF‐κB and TLR2/4‐MyD88/TRIF‐MAPK signaling pathways in IBPS‐3A‐induced macrophage M1 polarization. Collectively, these findings indicate that IBPS‐3A exerts its immunostimulatory effects probably through TLR2 and TLR4, subsequently triggering the NF‐κB and MAPK signaling pathways to induce macrophage M1 polarization.

## 4 Discussion

As crucial components of plant cell walls, pectic polysaccharides are widely recognized for their diverse biological activities, particularly their immunomodulatory potential (Kassab 2025). However, pectins from different botanical sources often exhibit distinct activities and mechanisms of action owing to variations in their fine structures. In this study, a pectin polysaccharide, IBPS‐3A, from the sweet potato vines was first extracted, purified, and systematically characterized. Furthermore, we explored the mechanisms underlying its immunomodulatory effects by inducing macrophage M1 polarization.

IBPS‐3A has a molecular weight of approximately 56 kDa. Structural analysis identified it as an acidic heteropolysaccharide consisting of a backbone primarily composed of 2‐α‐L‐rhamnose and 4‐α‐D‐galacturonic acid, with the rhamnose residues substituted at O‐4 by arabinans and galactans. This detailed structural characterization lays the foundation for further exploration of its potential as an immunomodulatory agent.

The balance between M1/M2 macrophage polarization plays a critical role in innate immune surveillance (Boutilier and Elsawa 2021). IBPS‐3A dose‐dependently induced M1 polarization in RAW264.7 macrophages, as evidenced by morphological changes characteristic of an activated state and increased cell proliferation in a concentration‐dependent manner; significant increases in the secretion of pro‐inflammatory cytokines TNF‐α and IL‐6, and production of the key effector molecule nitric oxide (NO), while upregulating the M1 marker protein iNOS and downregulating the M2 marker protein Arg‐1. These findings are consistent with the immunostimulatory effects reported for other dietary pectins, such as those from *C. pilosula* (Zou et al. 2014) and *Citrus aurantium* (Liu, Huang, et al. 2025).

Notably, although IBPS‐3A itself exhibited no direct cytotoxicity against B16 melanoma cells (Figure 6A), the conditioned medium from IBPS‐3A‐pretreated macrophages effectively inhibited B16 cell proliferation. This result suggests that the antiproliferative effect is indirectly mediated through immune system activation rather than through direct cytotoxicity. To further validate this in a model that more closely mimics a cellular interaction scenario, a Transwell co‐culture system was established. Coculture with IBPS‐3A‐activated macrophages significantly induced B16 cell death in a dose‐ and cell density‐dependent manner (Figure 7). These findings demonstrate that IBPS‐3A primes macrophages to exert significant effector functions, underscoring its potential as an immunomodulatory agent.

To elucidate the potential molecular targets by which IBPS‐3A interacts with immune cells, a preliminary mechanistic investigation was conducted to assess its mode of action as a dietary component. Toll‐like receptors (TLRs) are key recognition receptors for dietary polysaccharides in the gut‐associated lymphoid tissue (GALT) (Son et al. 2022). Previous studies have shown that sulfated *Cyclocarya paliurus* polysaccharides can activate macrophages through TLR4‐mediated MAPK/NF‐κB signaling (Yu et al. 2024), while recent evidence further supports TLR4 as an important target in the immunoregulatory effects of sulfated *C. paliurus* polysaccharides (Chen et al. 2026). In the present study, molecular docking calculations predicted that IBPS‐3A has potential binding interactions with TLR2/4. Moreover, inhibitor experiments revealed that a TLR4‐specific inhibitor strongly suppressed IBPS‐3A‐induced production of inflammatory factors, whereas a TLR2 inhibitor showed a partial inhibitory effect. The combined use of both inhibitors resulted in maximal suppression (Figure 8). This suggests that TLR4 may be the primary functional receptor for IBPS‐3A and that TLR2 potentially plays a synergistic or auxiliary role in its recognition. Furthermore, Western blot analysis demonstrated that IBPS‐3A markedly increased the phosphorylation levels of p65 (NF‐κB pathway) and p38 (MAPK pathway), and this activation was blocked by TLR2/4 inhibitors (Figure 8J,K). Collectively, these data indicate that IBPS‐3A likely binds to TLR4 (and TLR2) on macrophage surfaces, initiates downstream MyD88/TRIF‐dependent signaling, activates the NF‐κB and p38 MAPK pathways, and ultimately drives M1 polarization (Figure 8L).

Furthermore, the complex neutral sugar side chains within the RG‐I region—particularly the highly branched arabinogalactan rich in (1 → 5)‐α‐L‐Ara*f* linkages—may serve as the critical “signal tag” for efficient activation of TLR2/4 signaling. Wu et al. (2026) revealed that the immunostimulatory effect of a pectic polysaccharide (AEFP‐B1) from *Aralia elata* (Miq.) Seem., which similarly possesses a backbone comprising HG and RG‐I domains, was directly attributed to the glucuronoarabinogalactooligosaccharide side chains of its RG‐I domain, These side chains bound to and activated TLR2 and TLR4 with high affinity. Similarly, Son et al. (2022) demonstrated that REP‐I, an RG‐I‐type pectic polysaccharide from radish leaves, is capable of being recognized by several pattern recognition receptors on macrophages (e.g., Dectin‐1, scavenger receptors, TLR2, and TLR4), differentially activating downstream JNK/ERK/NF‐κB pathways. Given that IBPS‐3A possesses similar arabinogalactan side chains in its RG‐I region, it is reasonable to propose that IBPS‐3A follows a mechanism comparable to that of immune recognition. This study lays the foundation for further exploration of the immunomodulatory mechanisms of sweet potato vine pectic polysaccharides and their associated signaling pathways.

## 5 Conclusion

This study successfully isolated and purified the polysaccharide IBPS‐3A from sweet potato vines. IBPS‐3A has a molecular weight of approximately 56 kDa and is characterized as an acidic heteropolysaccharide, consisting of a backbone primarily composed of 2‐α‐L‐rhamnose and 4‐α‐D‐galacturonic acid, and the rhamnose residues are substituted at *O*‐4 by arabinans and galactans. We report for the first time that this polysaccharide possesses potent immunomodulatory activity, specifically inducing macrophage polarization toward the M1 phenotype via TLR2/4‐mediated NF‐κB/p38 MAPK activation. This activation leads to a marked, macrophage‐dependent inhibition of tumor cell proliferation, confirming the functional potency of IBPS‐3A as an immunostimulatory agent. This study highlights the potential use of sweet potato vine polysaccharides as a functional food for immunomodulation.

## Author Contributions

**Yuqi Shi:** writing – original draft, investigation, methodology. **Bo Si:** data curation, formal analysis. **Yu Shi:** conceptualization. **Wenxin Geng:** writing – review and editing, software. **Dunchi Xiao:** formal analysis. **Zhixiang Yuan:** supervision, project administration, resources. **Liwen Li:** project administration, resources, supervision.

## Funding

This work was supported by the Science and Technology Commission of Shaanxi Province (no. 2020ZDLSF03‐06).

## Ethics Statement

This study does not involve any human or animal testing.

## Conflicts of Interest

The authors declare that they do not have any conflicts of interest. Changzhou Institute of Materia Medica Co. Ltd. has no commercial interest in sweet potato vine polysaccharide products.

## Supporting information


**Table S1:** Molar percentages of monosaccharide composition of IBPS‐3A.
**Figure S1:** Preliminary comparison of the effects of IBPS‐1, IBPS‐2 and IBPS‐3 on RAW264.7 macrophage polarization.
**Figure S2:** TIC chromatogram of the methylated alditol acetates of IBPS‐3A.
**Figure S3:** NMR spectrum of IBPS‐3A.
**Figure S4:** Western blot analysis of iNOS and Arg‐1 protein expression.
**Figure S5:** Cytotoxic effect of low‐concentration conditioned medium on B16 cells.
**Figure S6:** Cytotoxic effect of IBPS‐3A on B16 cells in a co‐culture system.
**Figure S7:** Molecular docking simulation diagram of the IBPS‐3A main chain with TLR2/4.

## Data Availability

The data that support the findings of this study are available from the corresponding author upon reasonable request.
