# Supplementary material for: Structural Characterization and Immunomodulatory Activity of a Polysaccharide From Ipomoea batatas L. Vine: Activation of Macrophage
Source: Food Sci Nutr. 2026 Nov 3;14(11):e72530. doi: 10.1002/fsn3.72530 (PMC13731730; doi:10.1002/fsn3.72530)
Supplement: Supplementary file 1 [file FSN3-14-e72530-s001.docx]

**Supplementary Material**

**Table S1**

Molar percentages of monosaccharide composition of IBPS-3A

| **Molar ratio (%)** | | | | |
| --- | --- | --- | --- | --- |
| Rha | GalA | Glc | Gal | Ara |
| 3.17 | 47.94 | 0.82 | 27.65 | 20.42 |

Fig. S1. Preliminary comparison of the effects of IBPS-1, IBPS-2 and IBPS-3 on RAW264.7 macrophage polarization.

Fig. S2. TIC chromatogram of the methylated alditol acetates of IBPS-3A.

Fig. S3. NMR spectrum of IBPS-3A.

(A) DEPT-135 NMR spectrum of IBPS-3A.

(B) HMBC (120-180 ppm) spectrum of IBPS-3A.

Fig. S4. Western blot analysis of iNOS and Arg-1 protein expression

(A) Representative Western blot bands for iNOS and Arg-1.
(B) Densitometric quantification of Western blot bands showing relative protein expression levels.

Fig. S5. Cytotoxic effect of low-concentration conditioned medium on B16 cells.

Fig. S6. Cytotoxic effect of IBPS-3A on B16 cells in a co-culture system.

Fig. S7. Molecular docking simulation diagram of the IBPS-3A main chain with TLR2/4.
